# Brain activation underlying turning in Parkinson’s disease patients with and without freezing of gait: a virtual reality fMRI study

**DOI:** 10.1038/npjparkd.2015.20

**Published:** 2015-10-22

**Authors:** Moran Gilat, James M Shine, Courtney C Walton, Claire O’Callaghan, Julie M Hall, Simon J G Lewis

**Affiliations:** 1 Parkinson’s Disease Research Clinic, Brain and Mind Research Institute, The University of Sydney, Sydney, NSW, Australia; 2 Department of Psychology, Stanford University, Stanford, CA, USA; 3 Department of Psychology, Behavioural and Clinical Neuroscience Institute, University of Cambridge, Cambridge, UK; 4 School of Social Sciences and Psychology, University of Western Sydney, Sydney, NSW, Australia

## Abstract

**Background:**

Freezing of gait is a debilitating symptom affecting many patients with Parkinson’s disease (PD), causing severe immobility and decreased quality of life. Turning is known to be the most common trigger for freezing and also causes the highest rates of falls. However, the pathophysiological basis for these effects is not well understood.

**Methods:**

This study used a virtual reality paradigm in combination with functional magnetic resonance imaging to explore the neural correlates underlying turning in 17 PD patients with freezing of gait (FOG) and 10 PD patients without FOG while off their dopaminergic medication. Participants used foot pedals to navigate a virtual environment, which allowed for blood oxygen level-dependent (BOLD) responses and footstep latencies to be compared between periods of straight “walking” and periods of turning through 90°. BOLD data were then analyzed using a mixed effects analysis.

**Results:**

Within group similarities revealed that overall, PD patients with freezing relied heavily on cortical control to enable effective stepping with increased visual cortex activation during turning. Between groups differences showed that when turning, patients with freezing preferentially activated inferior frontal regions that have been implicated in the recruitment of a putative stopping network. In addition, freezers failed to activate premotor and superior parietal cortices. Finally, increased task-based functional connectivity was found in subcortical regions associated with gait and stopping within the freezers group during turning.

**Conclusions:**

These findings suggest that an increased propensity towards stopping in combination with reduced sensorimotor integration may underlie the neurobiology of freezing of gait during turning.

## Introduction

Turning is an integral yet complex task of daily mobility that commonly precipitates falls in the elderly population.^1^ This effect is greater in Parkinson’s disease (PD),^2^ significantly increasing the risk of falls and related injuries, such as hip fractures^1^ leading to nursing home placement.

One reason for the increased incidence of falls in PD is known to be freezing of gait (FOG), which is described as a brief, episodic absence or marked reduction of forward progression of the feet despite the intention to walk.^3^ Importantly, turning is recognized to be the most frequent trigger of this phenomenon.^4^ This debilitating symptom impacts around half of PD patients, causing regular falls and a decreased quality of life (for review see Nutt *et al.*).^3^

Behavioral measures of turning difficulties and their association with FOG have been widely studied. For instance, patients with FOG turn more slowly, take more steps, are more variable in their step times and implement a different turning strategy when compared with PD patients without FOG and healthy controls.^2,5^ Turning difficulties in PD patients with FOG are only partly improved by dopaminergic medication^2^ and any amelioration achieved through cueing has only a short lasting carry over effect after cue removal.^6^

Due to difficulties inherent in the neuroimaging of gait, the pathophysiological mechanisms linking turning and freezing are currently poorly understood, limiting our ability to develop adequate therapeutic interventions. Recent insights have been gained from the effects of deep brain stimulation in the subthalamic nucleus (STN-DBS)^7^ and saccadic functioning^8^ in Parkinson’s disease. The STN, with its striatal, cerebellar and hyper-direct supplementary motor and other frontal cortex connections,^9–11^ is thought to be involved in a common neural pathway underlying FOG causing inhibition of the gait related subcortical structures.^12,13^ As turning is a provocative trigger for FOG, one might predict that abnormal STN activation by itself, or indirectly through abnormal activation in its cerebellar, striatal and frontal cortex connections is at least in part responsible for the deficits in turning kinematics seen in PD patients with FOG. Indeed, Lohnes and Earhart^7^ showed that STN-DBS in PD patients shortened their turn duration, whereas it also improved saccadic functions that are important for turning.^7^ STN-DBS has also been shown to improve visuospatial attention^14^ and decrease intersegmental latencies (e.g., eye–head, eye–foot, and head–trunk), which are both affected in PD patients, especially in those that experience freezing.^7,15^ However, though these studies provide valuable information regarding the STN’s role, little information exists about other regions of the brain that are likely to be involved in the functional impairments that cause turning difficulties and FOG in PD patients.^12^

It is therefore clear that novel paradigms are needed to improve our understanding of the pathophysiology underlying turning deficits in Parkinson’s disease, especially when exploring freezing of gait. As such, the current study set out to investigate the effect of turning during the performance of an interactive virtual reality (VR) paradigm that has previously been used in combination with functional magnetic resonance imaging (fMRI) to investigate the pathophysiology underlying behavioral freezing episodes^13^ and the effects of other known triggers of FOG, such as high cognitive load.^16,17^ However, the current study will be the first to combine an adjusted version of the VR with fMRI to explore the widespread brain regions that underpin turning behavior in Parkinson’s disease patients with and without freezing of gait. The current VR paradigm allows for the investigation of complex sensorimotor integration during turning, as it requires subjects to generate effective lower limb motor output while updating changes in their visual environment. We hypothesized that PD patients with FOG would be slower and more variable in their step times during the navigation of a turn in the VR^18,19^ and that turning would elicit altered activation patterns in the STN^7^ and its hyperdirect frontal cortex connections.^9–11^ In addition, we expected to find differences across the cortical, striatal and other subcortical regions that have previously been identified in PD patients with FOG during structural MRI,^11,20^ fMRI resting state,^21^ PET gait imagery tasks^22^ and during fMRI with a gait imagery tasks,^23,24^ upper limb motor task,^25^ and VR performance.^13,16,17^

## Materials and methods

### Patient details and study protocol

A total of 27 levodopa-responsive patients with idiopathic PD were recruited from the Parkinson’s Disease Research Clinic, Brain and Mind Research Institute, the University of Sydney having satisfied UKPDS Brain Bank clinical diagnostic criteria. Seventeen Parkinson’s disease patients with freezing of gait (PD+FOG) were selected based on a previously obtained positive score on the Freezing of Gait Questionnaire question 3 (FOG-Q3)^26^ (“do you feel that your feet get glued to the floor while walking, making a turn or when trying to initiate walking (freezing)?”) and 10 non-freezing Parkinson’s disease− patients (PD−NF) were selected based on a null score on this question. All patients underwent neurological and neuropsychological assessment, completed a gait protocol and performed an fMRI scanning session in their practically defined ‘off’ state, having been withdrawn from dopaminergic medication overnight for more than 12 h before testing. The patients in the current study were measured “off” their dopaminergic medications to increase the likelihood of eliciting freezing like behaviors in a standardized environment, to allow us to better investigate dopamine dependent basal ganglia dysfunctions and finally, to enable fair comparisons with previous fMRI studies.^13,23,24^ Ethical approval for this study was obtained from the University of Sydney Human Research Ethics Committee and written informed consent was obtained from each patient.

### Neurological and cognitive assessment

All patients were assessed on the motor section of the Unified Parkinson’s Disease Rating Scale (UPDRS-III) and Hoehn and Yahr Stage in their “off” state. In addition, the Mini Mental State Examination, Montreal Cognitive Assessment, and Hospital Anxiety and Depression Scale (HADS) were obtained and their daily Dopamine Dose Equivalency was calculated.

### Gait protocol

Each subject completed eight video recorded 5-m long timed up and go tasks, during which each turn was performed inside a 50-cm^2^ taped box on the ground. The timed up and go tasks incorporated 180 and 540° turns in both directions, taking short steps around the outline of the box in both directions and two vocal dual tasks (i.e., naming multiples of nine and months of the year backwards) with 180° turns in both directions. The videos were scored offline for periods of freezing as defined by a brief, episodic absence or marked reduction of forward progression of the feet despite the intention to walk.^3^ The timed up and go tasks clinically confirmed FOG in all but one subjects in the PD+FOG group, whereas none of the PD−NF patients experienced any freezing episode. The one subject in the PD+FOG group was still included into the study based on a positive score on the FOG-Q3 and UPDRS question 3.11 and because he was seen by an experienced physician to have experienced freezing when arriving into the clinic.

### Virtual reality paradigm

**Figure 1:**
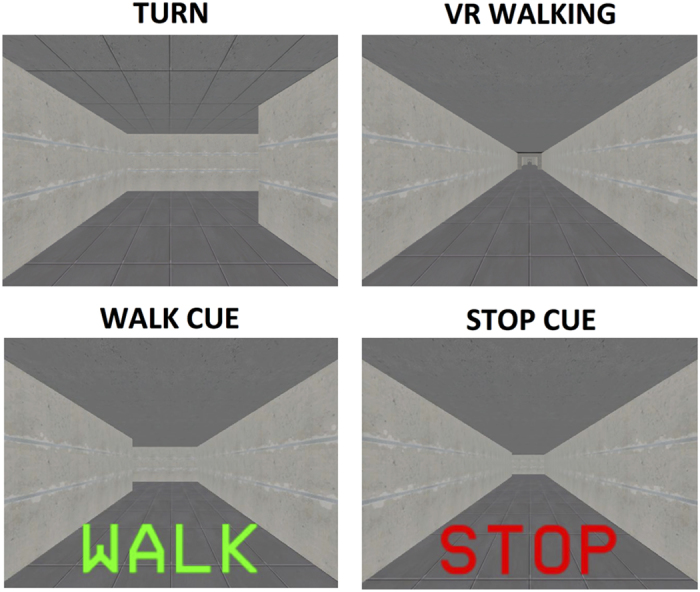
Representation of the virtual reality task showing a turn, a period of virtual reality walking and a WALK and STOP cue. WALK, walking.

Patients performed the VR while lying inside the fMRI scanner. The task took ~ 6 min to complete and was presented on a screen that could be clearly viewed via a mirror mounted onto the head coil. The virtual environment was a three-dimensional corridor presented in the first-person. Forward progression through this corridor was accomplished by alternately depressing left and right foot pedals at least 30° below parallel in a “physiological” sequence (e.g., left–right). Out of sequence steps (left–left or right–right) did not result in forward progression and were disregarded from the analyses. Patients were instructed to tap the pedals in a comfortable rhythm. The VR only contained turns and simple “STOP” cues presented in the color red, followed by a simple “WALK” cues presented in the color green (Figure 1), which were added to ensure that the patients were still paying attention to the task. No other environmental triggers (e.g., doorways) or complex cognitive cues were presented during this experiment in distinction to our previous reports.^13^ The turns in the VR were 90° and randomly presented in both directions (Figure 1). An average of 23 turns were presented during the trial and patients had to take between 3 and 6 steps to complete a turn, based on their stepping latencies. The VR automatically presented the navigation of a turn as a reaction to the physiological sequence of foot pedal depressions. We chose to leave out an additional motor task to prevent dual tasking from inducing any freezing.^27^ As such, no difference in behavioral motor activation was required between periods of straight walking and periods of turning, except for potential eye movements induced by the updating of visual information as the turn was presented on the screen. Similar to previous studies,^2,24^ no distinction was made between left and right turns in order to increase the power of the analyses.

### Behavioral measures

Footstep latencies were calculated by measuring the time between two consecutive foot pedal depressions. The mean and standard deviation were then used to calculate the coefficient of variation for the three steps that were taken during a turn and for three randomly selected steps that were unrelated to any turn or “STOP” and “WALK” cues (hereafter defined as “walking”). In addition, we calculated the longest footstep latency in those three steps (defined as the maximum footstep latency). Any maximum footstep latency that was greater than twice the modal footstep latency was considered to be a behavioral freezing episode, as described in more detail elsewhere.^13,19^ All behavioral freezing episodes were removed from the current analyses to ensure that any results in this study were owing to the effect of turning and were not being driven by the occurrence of any freezing episodes.^17^

### Neuroimaging

#### Event related analysis

The image acquisition and image preprocessing steps are described elsewhere.^17^ Individual first-level spatial maps were created in Statistical parametric mapping software (SPM8, Wellcome Trust Centre for Neuroimaging, London, UK, http://www.fil.ion.ucl.ac.uk/spm/software/) using a general linear model analysis within an epoch-related design in a fixed-effects analysis. A design matrix was created for each patient by entering two regressors for each trial, namely, a regressor that modeled the specific onset times and associated temporal derivatives for each turn and a regressor that similarly modeled periods of walking. The walking epochs were randomly selected and scaled to the number of turning epochs, both covering the total duration of the task to control for possible effects of fatigue. All patients were instructed to minimize head motion by only moving the ankles, while not raising the legs and preventing hip rotation. In addition, a brief trial run was performed inside the scanner before the start of the task. This allowed a researcher to adjust the position of the patient’s feet and give additional instructions if extensive head motion was detected. This, together with the placement of cushions inside the head coil ensured optimal performance with the least amount of head motion. After data collection, any trial with >3-mm head motion was excluded from the analyses and six motion and nuisance regressors were added into the first level analysis per subject, controlling for movement artifacts in the three directions of translation and axes of rotation. Contrast images from the first-level analyses were then entered into a second-level random-effects independent samples *t*-test design analysis to determine the group differences on the contrast of interest (turning>walking). This contrast was chosen as it minimizes the differences between the two conditions, both requiring bi-pedaling motor output while watching a screen, with the only difference being going through a turn. It therefore controls for the variance associated with bi-pedaling motor output and watching a screen, while allowing the resultant brain activation pattern to be interpreted as the effects associated with turning. HADS anxiety, HADS depression and Montreal Cognitive Assessment scores were entered as covariates at the second level. Whole brain voxel maps were displayed using XjView (www.alivelearn.net/xjview) software (*P*<0.005). To decrease the risk of type-II error, we used a large cluster size threshold (*k*>20 voxels).^28^

#### Region of interest analysis

Spherical 8-mm regions of interest (ROI) were drawn around the peak voxels from the second level T-map by using the MarsBar toolbox in SPM8 (ref. 29). The peak voxel values and their coordinates are presented in Table 2. Importantly, these values were not used for any further statistical inference, but instead allowed us to further explore the linear direction of blood oxygen level-dependent (BOLD) response patterns found in the whole brain analysis, as described elsewhere.^17^

In addition, previous studies have implicated key striatal and subcortical regions in turning and behavioral freezing in patients with Parkinson’s disease.^7,11,23,24^ As such, we subsequently explored the images from the first-level analysis using predefined regions of interest, which were analyzed independently from the whole brain analyses. Spherical ROI’s were drawn around the following left and right striatal regions: caudate nucleus, putamen and ventral striatum, and subcortical regions: mesencephalic locomotor regions (MLR), globus pallidus internus (GPi), STN and the bilateral cerebellar locomotor region (CLR; see Supplementary Table 1 for coordinates in Montreal Neurological Institute (MNI) space). MarsBar^29^ was used to extract percent signal change values for each region and a difference score was calculated between periods of turning and periods of walking. Two-sided independent sampled *t*-tests were performed on the group level and paired sampled *t*-tests were used within groups. Alpha levels were set to 0.05.

#### Task-based functional connectivity

On the basis of current perceptions that FOG is likely due to functional network deficits,^12,16^ we aimed to explore the task based functional connectivity patterns^30^ associated with turning in the VR. As such, the Marsbar toolbox was also used to extract raw ROI data (beta weights; or β) of each ROI for each patient. MATLAB (The MathWorks Inc., Natick, MA, USA) was used to calculate the temporal derivative (TD=β (T_n_)—β (T_n-1_)) of the raw β weights for each ROI. After scaling each time course by its variance, the temporal derivatives of each ROI were multiplied by the temporal derivatives of the other ROI’s for each time point, such that a positive score reflected ‘functional coupling’ between a pair of ROIs. For each subject, we multiplied the functional coupling score for each temporal derivative by the convolved time points associated with either turning or walking periods in the virtual reality task. We then calculated the non-zero average for each ROI pair for both contrasts for each patient. Paired sampled *t*-tests were employed to analyze the differences in these non-zero average scores between periods of turning and periods of walking. Finally, the non-zero average scores were organized into two 13-by-13 matrices for each subject, one for the periods of turning and one for the periods of walking for each of the 13 ROI’s. These matrices were then compared statistically using the Network Based Statistics Toolbox^31^ with a threshold value of 3.0, *P*<0.05 as well as the False Detection Rate option (*P*<0.05) in the network-based statistics software to control for multiple comparisons.

## Results

### Patient demographics

The demographic statistics, results of the gait assessments and the behavioral measures from the VR are presented in Table 1. The groups were matched for key demographics such as age, disease duration, dopamine dose equivalency, mini mental state examination scores, disease severity and motor severity (UPDRS-III) after removing the gait and freezing items. Moreover, both groups included more males (*χ* ^2^(1)=0.764, *P*=0.382). As is commonly reported, the PD+FOG group did have significantly lower Montreal Cognitive Assessment scores and higher HADS anxiety and HADS depression scores (Table 1).^17^ The current study therefore controlled for the significant group differences in Montreal Cognitive Assessment and HADS scores through the use of covariate analyses. Additional non-parametric analyses revealed that the groups were also matched for Hoehn and Yahr stages and that only the PD+FOG group scored positively on the FOG-Q3 (Table 1). Finally, 53% of the PD+FOG group and 60% of the PD−NF group (*χ* ^2^(1)=0.127, *P*=0.722) had worse Parkinson’s disease symptoms on the left side of the body as calculated by a ratio of the sum of UPDRS-III items related to symptom severity on the right side and left side of the body. Finally, no significant differences were found between the groups on handedness (*χ* ^2^(1)=1.27, *P*=0.260), foot tapping abilities and leg agility as obtained by the UPDRS questions 3.7 and 3.8, respectively (Table 1). In addition, toe tapping and leg agility scores did not correlate with the behavioral measures of the VR task (results not shown).

### Gait assessment

Turning indeed proved to be a provocative trigger for FOG, as 76% of the patients in the PD+FOG group froze during the 540° turns and 41% froze during the 180° turns. In addition, only 35% of the patients froze when having to perform a cognitive dual task during straight walking while 71% froze when dual tasking was performed during the performance of a 180° turn (Table 1). PD−NF patients did not experience any freezing. There was no significant difference in the amount of freezing experienced by PD+FOG patients between left and right turns (540**°**: *t*=1.541, *P*=0.143 and 180**°**: *t*=0.490, *P*=0.631).

### Virtual reality task

Turning during the VR provoked behavioral freezing episodes in 10 PD+FOG patients with an average of 13% of turns eliciting a freeze in those patients, whereas none of the PD−NF patients experienced a behavioral freeze during turning (*U*=35, *Z*=−2.793, *P*<0.01). In addition, even when removing all behavioral freezing episodes, PD+FOG still had significantly higher scaled maximum footstep latencies when turning compared with walking (*t*(16)=2.17, *P*=0.045), whereas PD−NF had similar scaled maximum footstep latencies (*t*(9)=0.693, *P*=0.506). As predicted PD+FOG also had higher step time variability compared with the non-freezer group as shown by an increased coefficient of variation during both turning and walking (Table 1).^19^ Within groups, the coefficient of variation was slightly higher but not significantly different during turning compared with walking (PD+FOG: *t*=0.815, *P*=0.427, PD−NF: *t*=0.437, *P*=0.672). It is important to note that although PD+FOG patients had a slightly higher cadence, no significant differences in modal footstep latencies were found between the groups, which together with similar UPDRS-III questions 3.7 (toe tapping) and 3.8 (leg agility) scores, indicates that any group differences found during turning were unlikely to be due to an overall difference in motor performance.

### Neuroimaging results

#### Within-group similarities

**Figure 2:**
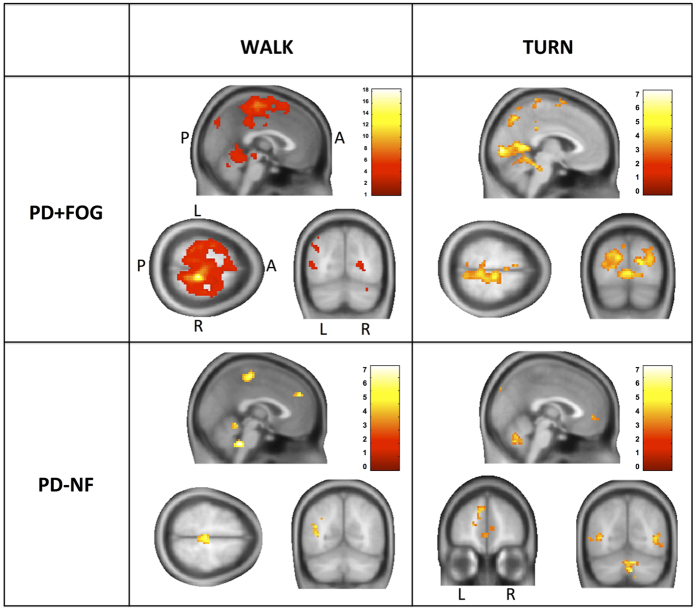
Within-group whole brain results for periods of walking (WALK) and turning (TURN) in the virtual reality task. A, anterior, L, left; P, posterior, R, right; *k*>20 and *P*<0.005.

**Figure 3:**
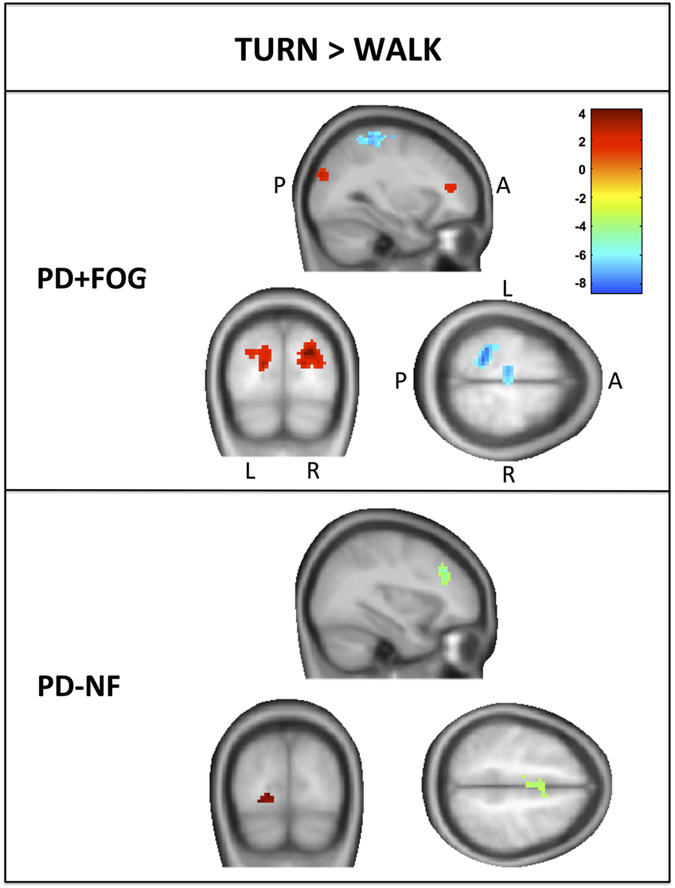
Within-group whole brain results for the contrast turning>walking in the virtual reality task. A, anterior, L, left; P, posterior, R, right; *k*>20 and *P*<0.005.

Figure 2 shows the whole brain BOLD response patterns for periods of walking and turning for each group separately. The results of the within group turning>walking contrasts are presented in Figure 3. The PD+FOG group required widespread activation across the motor and visual cortices, cerebellum, and MLR region to achieve stepping in the virtual reality task when compared with the selective cortical recruitment in the PD−NF group. The PD+FOG group also activated the cerebellum during turning, but this time with a more caudal region of the medulla. PD−NF required less activation across the motor, visual and cerebellar cortices, while recruiting more medial frontal regions.

#### Between group differences

**Figure 4:**
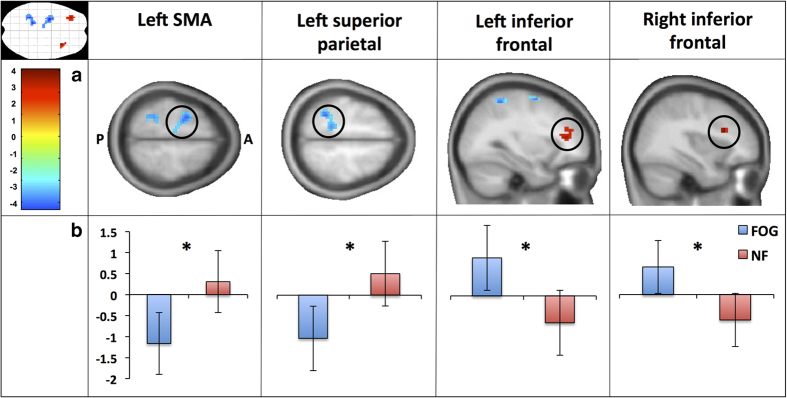
Results of the whole brain second level group effect for turning>walking in the virtual reality task. (**a**) Screenshots from the whole brain second level independent *t*-test for the turning >walking contrast showing PD+FOG>PD−NF results (P=Posterior, A=Anterior, k>20,*P*<0.005, uncorrected, see Table 2), while using MOCA and HADS scores as covariates and. (**b**) The average beta intensities of the peak voxel 8 mm spherical ROI’s for the turning >walking contrast in the virtual reality task for both Parkinson’s disease patients with freezing of gait (FOG) and without freezing of gait (NF). HADS, Hospital Anxiety and Depression Scale; MOCA, Montreal Cognitive Assessment; PD+FOG, Parkinson’s disease patients with freezing of gait; PD−NF, Parkinson’s disease patients without freezing of gait; ROI, regions of interest.

Comparing the groups for the contrast of turning>walking revealed four brain areas with significantly different BOLD responses (Figure 4). PD+FOG showed decreased BOLD responses across the left supplementary motor area (SMA) extending to the left premotor area and the left superior parietal lobule with increased BOLD responses in both the left and right inferior frontal gyrus when compared with PD−NF. Peak voxel statistics and coordinates are presented in Table 2. Although the left superior parietal lobule was significantly different with the current statistical settings, the right superior parietal lobule also appeared as to have decreased BOLD activation during turning in PD+FOG compared with PD−NF when lowering the cluster size threshold to 13 voxels (*t*=−3.08, *P*=0.003).

#### Predefined ROI analysis

No significant group differences were found for percent signal changes of striatal and subcortical ROI’s when contrasting turning with walking in the VR. However, within the PD+FOG group a significant increased percent signal change in the left caudate nucleus (*t*_16_=2.75, *P*=0.014) was found in the difference score between turning and walking, whereas no differences were found for PD−NF. The right caudate also showed increased percent signal change for the PD+FOG group, but this did not reach statistical significance (*t*_16_=1.72, *P*=0.106). Finally, a supplementary analysis using a predefined spherical ROI of the pre-SMA (MNI: −3; 6; 53)^32^ revealed reduced activation, although not significantly different, in the PD+FOG group during turning compared with walking in the VR (Average beta: −0.664, *t*=1.55, *P*=0.138).

#### Task-based functional connectivity

**Figure 5:**
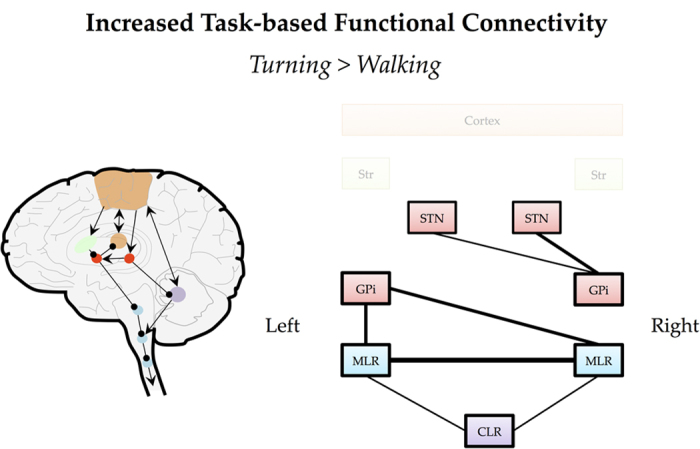
Representation of the task based functional connectivity results. Figure shows the comparison between periods of turning and periods of walking in the virtual reality task within the Parkinson’s disease patients with freezing of gait group. CLR, cerebellar locomotor region; GPi, globus pallidus internus; MLR, mesencephalic locomotor region; STN, subthalamic nucleus.

Four highly significant functional connectivity scores survived network-based statistics correction^31^ with stringent threshold settings (threshold=3.0, *P*=0.007) when comparing periods of turning with walking within the PD+FOG group (Figure 5). Specifically, we observed increased connectivity between the bilateral MLR (*t*=5.16, *P*<0.001), between the left GPi and both the right (*t*=4.14, *P*<0.001) and the left (*t*=3.53, *P*<0.01) MLR and finally, between the right GPi and the left STN (*t*=3.32, *P*<0.01). At a lower statistical threshold of 2.5 (*P*=0.023), additional significant functional connectivity scores were revealed between the right GPi and the right STN (*t*=2.83, *P*=0.01) and between the bilateral CLR with both the left (*t*=2.86, *P*<0.01) and right MLR (*t*=2.84, *P*=0.01). The increased functional connectivity score between the bilateral MLR remained significant (*P*<0.05) when using the false detection rate option in the network-based statistics software. No changes in functional connectivity were found within the PD−NF group between periods of turning and walking. In addition, no significant group differences in functional connectivity were found that survived network based statistic or false detection rate correction.

## Discussion

This is the first study to investigate brain activation patterns underlying turning during a virtual reality task in Parkinson’s disease patients with and without freezing of gait. Within group results revealed that PD+FOG relied heavily on widespread cortical control of their movements, whereas PD−NF achieved more successful stepping with a selective motor network. Between group results showed that during turning, patients with freezing of gait displayed increased BOLD responses in bilateral inferior frontal regions and decreased BOLD across the left premotor cortex and left superior parietal cortex when compared with PD−NF. In addition, PD+FOG showed increased percent signal changes in the left caudate nucleus and displayed strong functional connectivity between the GPi, STN, cerebellar and mesencephalic locomotor regions during turning relative to walking. Importantly, an additional analysis without the subject in the PD+FOG group that did not freeze during the gait assessment (*n*=16) revealed the same brain activation patterns, aiding towards the robustness of our findings.

Turning during walking is a difficult motor task to investigate using current neuroimaging techniques. Most evidence regarding the effects of turning in PD patients with FOG therefore comes from indirect and spatially limited techniques, such as the effect of STN-DBS, transcranial magnetic stimulation and the effects of dopaminergic medication.^2,7,33^ Thus far only one study has used fMRI to investigate the effects of turning in PD patients with FOG. Peterson *et al.*^24^ used gait imagery of simple (forward) and complex (backward or turning) movements in combination with fMRI to report BOLD responses of five locomotor regions of interest in PD patients with and without FOG.^24^ They found that for PD patients without FOG, gait imagery of simple forward walking compared with rest was associated with increased BOLD responses in several locomotor regions, including the SMA. However, patients with FOG actually showed decreased activation in the globus pallidus and mesencephalic locomotor region with trends towards decreased activation in the right SMA.^24^ However, despite the large clinical impact turning has on freezing, no significant differences were found in that study between imagined forward walking and turning in PD patients with and without FOG. In addition, none of the patients in that study reported freezing during imagined gait.^24^ This indicates that the complexity between the gait imagery tasks might be too subtle to induce detectable changes in brain activation within a Parkinson’s disease cohort.^24^ These methods may also have lacked key challenges in sensorimotor integration associated with turning and freezing of gait.^34–36^ As such, the current study set out to investigate turning-related brain activation by using an interactive virtual reality task that required patients to perform a lower limb motor task while having to update a changing visual environment similar to turning.

### Within groups: walking

The within-group similarities during periods of walking revealed that when off medication, PD+FOG patients required widespread activation across their motor cortices, cerebellum and MLR region, whereas PD−NF used focused activation patterns across the motor cortex, frontal cortex, and cerebellum to perform the task. The widespread cortical involvement seen in the PD+FOG group might indicate that even during simple forward walking in the VR, these patients are unable to rely on lower-order automatic behaviors and therefore must employ a more cortically controlled locomotor network.^3,11,12,37^ Indeed, we found that PD+FOG were unable to keep a consistent rhythm between their steps, as indicated by high step time variability scores, when compared with PD−NF. This result is consistent with previous studies looking at virtual reality task performance^19^ and over ground walking^18^ and is at least in part indicative to a loss of automaticity in locomotion, as stride time variability has been shown to improve with dopaminergic medication while worsening during dual tasking,^18,38^ indicating an ineffective basal ganglia involvement.^3,39^ Our findings also support the notion that although FOG is a paroxysmal phenomenon, patients with freezing already have altered brain activation patterns during effective walking.^18^ This could increase their susceptibility to freeze when additional information from competing pathways (e.g., sensorimotor, cognitive, and limbic) require concurrent processing by the basal ganglia causing response conflict that eventually presents itself as FOG.^12^

### Within groups: turning

Within group similarities during periods of turning in the VR revealed that PD+FOG relied more heavily on visual information while decreasing the recruitment of medial motor and left parietal cortices. This might indicate that the PD+FOG were over-reliant on visual information, possibly as a learned response to poor kinesthetic feedback.^35,40–42^ Turning in the VR might worsen this effect by preventing the subjects from actually rotating their bodies according to the visually presented turn, altering their expected kinesthetic feedback as associated with turning over ground.

Alternatively, the extra visuo-parietal activation seen in the PD+FOG group could reflect saccadic abnormalities that could lead to unsuccessful sensorimotor integration.^8,43,44^ Indeed, we also observed an increased percent signal change during turning in the caudate nucleus for the PD+FOG group, an area implicated in saccadic functioning.^45^ Because of strong functional interactions between the oculomotor system and basal ganglia circuitry,^46^ any inappropriate visuomotor integration could cause response conflict between predicted and actual motor outcomes, thus potentially eliciting FOG.^12^ Indeed, the failure to recruit medial motor and medial frontal regions in the PD+FOG group during turning could indicate a difficulty with facilitating internally driven motor actions when visual support falls away.^47^ This notion is supported by the fact that externally driven motor actions, such as achieved through visual cueing techniques can alleviate freezing of gait,^48^ whereas turning is the most provocative trigger for freezing of gait.^27^ Future studies are now needed to confirm saccadic dysfunctions during turning in the VR and to determine whether dopaminergic medication improves the basal ganglia circuitry during turning and thus visuomotor integration.^49^

The neural correlates underlying behavioral freezing episodes in a similar VR task are described in detail elsewhere.^13,16,17^ Such an analysis was not performed in the current study owing to the limited amount of behavioral freezing episodes recorded. The small amount of freezing episodes can partly be explained by the turns being 90°, with previous studies showing that sharper turns are more likely to cause FOG.^5,27^ Future studies are therefore encouraged to implement sharper turns in virtual reality tasks to increase the likelihood of eliciting FOG, allowing for those episodes to be modeled with sufficient power when using fMRI. In addition, studies are encouraged to use ambulatory electroencephalography systems to provide information about cortical activation underlying other critical sensorimotor challenges associated with turning (e.g., balance, changing step lengths, and posture) that could not be modeled in the current study. Finally, no objective measures were obtained during the current gait tasks, which were solely implemented to ensure accurate group allocations. Future studies using objective kinematical measures during turning are therefore needed to test whether the VR and neuroimaging findings presented here are indeed related to the deficits seen during over ground turning.

### Between groups: turning>walking

Between group differences showed that navigating a virtual turn caused PD+FOG to recruit similar regions as those associated with the putative “stopping network”^10^ that has also been implicated in detecting salient stimuli and attentional processes.^50^ In addition, strong functional connectivity patterns were found in the PD+FOG group during turning between subcortical regions and the STN, which has neural projections to the regions of this stopping network.^10^ The stopping network is most commonly implicated through stop-signal tasks, showing activation of the right inferior frontal gyrus together with the pre-SMA, which is both functionally and structurally connected with the inferior frontal gyrus.^10^ This network implements inhibition through the STN, which regulates its effects via the Substantia Nigra pars reticulata, leading to thalamic inhibition when stopping is successful.^10^ Although little is known regarding the involvement of this stopping network across a broader range of motor tasks, a generalizability of the brain’s network for simple stop signal tasks has been shown to exist in more ecologically valid scenarios.^51^ In addition, the regions of this network have already been implicated in several pathophysiological hypotheses of FOG.^11,12,39,52^ The recruitment of this braking network could be an adaptive brain function that arose during the gradual development of FOG as a result of fear of falling^53^ and difficulties navigating challenging environments.^35^ If so, this braking network might be over recruited during such locomotor challenges, precipitating more FOG.

A recent pathophysiological model suggests that the main output structures of the basal ganglia (i.e., GPi and Substantia Nigra pars reticulata) provide tonic GABAergic inhibitory tone over the brainstem structures that control gait (such as the MLR and dorsal pendunculopontine nucleus) and the motor thalamus, preventing any unwanted movements at rest.^12^ Importantly, this tonic inhibition can be deactivated when an appropriate motor plan from the motor cortex is processed by the basal ganglia, relieving this inhibitory output.^12^ However, one can argue that when patients with PD and FOG proactively recruit a cortically controlled braking network as described above, additional activation of the STN might overrule any inhibitory relief accomplished through the basal ganglia system. Furthermore, in patients with PD and FOG the motor plans presented might already be inappropriate due to abnormal sensory integration,^34^ especially during challenging situations,^42^ which together with a dopamine depleted basal ganglia system decreases the likelihood of successful cortico-basal relief of gait inhibition.

The current study also found increased BOLD responses in the PD+FOG group across the inferior frontal gyrus bilaterally along with increased functional connectivity among subcortical regions associated with stopping (e.g., STN and GPi) and movement (e.g., MLR and CLR). Importantly however, another key region of the stopping network, namely the pre-SMA, was absent while the left premotor area actually showed significant decreased activation patterns in PD+FOG. Indeed, a predefined ROI analysis of the pre-SMA revealed reduced activation, although not significantly different, in the PD+FOG group during turning compared walking in the VR. The lack of significant group differences across the basal ganglia, thalamic and subcortical regions associated with stopping can potentially be explained by the removal of all behavioral freezing episodes from the analyses. As such, our results are aligned with previous studies that also showed reduced premotor activation in PD patient with FOG during gait imagery^23,24^ and behavioral freezing in the virtual reality task,^13^ along with profound structural^20,54^ and similarly increased functional connectivity impairments^11^ that have been reported in PD+FOG between the (pre-) SMA, STN and the CLR and MLR.

Based on the current results it can be speculated that PD patients with FOG have an inherent over activity across a stopping network when turning and that during this time the pre-SMA fails to provide necessary contextual input, rendering the striatum unable to correctly update the ongoing motor plan.^11,52^ This forces the GPi and STN to “shut down” activity in its’ efferent targets through the synchronization of the MLR and CLR,^12^ which in turn prevents the movement centers of the brain (such as the motor cortex, thalamus, and cerebellum) from receiving sensory information to adjust the gait cycle. To make matters worse, much of the sensory information gathered will already be inadequate, as PD+FOG have known proprioceptive, postural, saccadic and sensorimotor integration deficits,^34,41–43^ which are all essential elements for the successful execution of a turn. Indeed, reduced BOLD responses were found in the current study in both the left premotor cortex along with the left and right superior parietal lobule, which are thought to be involved in the prediction of somatosensory consequences of a motor plan and the integration of sensorimotor information.^23^ The movements during a turn will therefore no longer match their predictions, leading to increased response conflict, which together with the prospective recruitment of a braking network, explains why turning is so likely to trigger FOG.

## Conclusion

During the navigation of a turn in the virtual reality task, Parkinson’s disease patients with freezing of gait show altered BOLD responses across regions that implicate the prospective recruitment of a stopping network, which may be manifested pathologically as a freeze when sensorimotor processing becomes more complex.

## Figures and Tables

**Table 1:** Demographic statistics and behavioral results

	*PD+FOG (*n*=17)*		*PD−NF (*n*=10)*			
*Demographics*	*Mean*	*s.d.*	*Mean*	*s.d.*	T-*value*	P-*value*
Age	67.4	6.2	64.8	4.1	1.15	0.262
Disease duration	116	63	92.4	28	1.09	0.285
DDE	776	321	785	296	0.07	0.945
UPDRS-III	37.2	12	30.1	11	1.53	0.139
MMSE	28.0	2.2	29.4	0.7	1.96	0.061
MOCA	25.6	3.2	28.3	2.3	2.36	0.027
HADS (Anxiety)	6.12	3.3	3.40	2.4	2.29	0.031
HADS (Depression)	5.59	2.6	1.40	1.7	4.53	<0.01
	Median	Range	Median	Range	Z-*value*	P-*value*
H&Y^a^	2.5	2.0–3.0	2.0	2.0–2.5	−1.55	0.122
FOG-Q3^a^	3.10	2.0–4.0	0	0	−4.14	<0.01
UPDRS 3.7a (Toe tap R)^a^	2.0	0–3.0	1.0	0–2.0	−1.70	0.127
UPDRS 3.7b (Toe tap L)^a^	2.0	0–3.0	1.0	0–2.0	−1.13	0.286
UPDRS 3.8a (Leg agility R)^a^	1.0	0–3.0	1.0	0–2.0	−1.67	0.127
UPDRS 3.8b (Leg agility L)^a^	1.0	0–3.0	1.0	0–2.0	−1.33	0.223
Virtual Reality task	Mean	s.d.	Mean	s.d.	T-*value*	P-*value*
Max SFSL turning	1.14	0.09	1.07	0.10	2.33	0.028
Max SFSL walking	1.12	0.09	1.06	0.02	2.34	0.027
CV turning	16.5	5.84	9.60	3.9	3.31	<0.01
CV walking	15.7	5.44	9.26	3.9	3.27	<0.01
Modal FSL	0.56	0.11	0.62	0.10	1.39	0.172

Abbreviations: PD+FOG, Parkinson’s disease patients with freezing of gait; PD−NF, Parkinson’s disease patients without freezing of gait.

Independent sample *t*-test results presented unless otherwise indicated.

Demographics: Disease duration given in months, DDE, daily dopamine dose equivalence; FOG-Q3, question 3 of the freezing of gait questionnaire; HADS, hospital anxiety and depression scale; H&Y, Hoehn and Yahr; MMSE, Mini Mental State Examination; MOCA, Montreal Cognitive Assessment; UPDRS-III, motor section of the unified Parkinson’s disease rating scale without Q10 (gait) and Q11 (freezing); UPDRS 3.7 and 3.8, Average scores on Q3.7a and Q3.7b and 3.8a and 3.8b of the UPDRS (Left and right toe tapping and leg agility, respectively). Virtual Reality task: CV, coefficient of variation; Max SFSL, maximum scaled footstep latency as scaled to the modal FSL; Modal FSL, modal footstep latency. All analyses were two-tailed with an alpha of 0.05.

a Mann–Whitney *U*-test used.

**Table 2:** Brain areas with significantly different BOLD responses in the second level using an independent *t*-test design for the contrast turning>walking in the virtual reality task, showing the PD+FOG>PD−NF peak voxel statistics

*Brain area*	x	y	z	*# voxels*	T-*value*	Z-*value*	P-*value*
L—SMA—Premotor	−21	−7	64	59	−4.17	3.54	<0.001
L—Parietal superior	−30	−46	58	57	−3.91	3.37	<0.001
L—Inferior frontal	−30	35	13	35	3.58	3.14	<0.002
R—Inferior frontal	36	20	25	20	4.04	3.46	<0.001

Abbreviations: BOLD, blood oxygen level-dependent; L—SMA, left supplementary motor area; PD+FOG, Parkinson’s disease patients with freezing of gait; PD−NF, Parkinson’s disease patients without freezing of gait; R, right.

*k*>20, *P*<0.005, uncorrected with coordinates in MNI space.
