# Supplementary material for: Brain activation underlying turning in Parkinson’s disease patients with and without freezing of gait: a virtual reality fMRI study
Source: NPJ Parkinsons Dis. 2015 Oct 22;1:15020–. doi: 10.1038/npjparkd.2015.20 (PMC5516618; doi:10.1038/npjparkd.2015.20)
Supplement: Supplementary Table 1 [file npjparkd201520-s1.doc]

**Supplementary Information**

Supplementary Table 1: MNI coordinates of predefined spherical ROIS

| **ROI** | **x** | **y** | **z** | **Radius (mm)** |
| --- | --- | --- | --- | --- |
| Caudate | ± 11 | 14 | 9 | 8 |
| Putamen | ± 28 | 3 | 6 | 6 |
| GPi | ± 14 | -2 | 3 | 4 |
| STN | ± 11 | -14 | -3 | 4 |
| VS | ± 9 | 9 | -8 | 5 |
| MLR | ± 7 | -27 | -18 | 6 |
| CLR | 0 | -49 | -20 | 8 |

NOTE: GPi = Globus Pallidus internus, STN=Subthalamic nucleus, VS=Ventral Striatum, MLR=Mesencephalic Locomotor Region, CLR=Cerebellar Locomotor Region.
